# Location and Gender Differences in Osteonecrosis of the Jaws in Patients Treated with Antiresorptive and Antineoplastic Drugs Undergoing Dentoalveolar Surgical, Systematic Review with Meta-Analysis and Trial Sequential Analysis

**DOI:** 10.3390/jcm12093299

**Published:** 2023-05-05

**Authors:** Mario Dioguardi, Francesca Spirito, Mario Alovisi, Riccardo Aiuto, Daniele Garcovich, Vito Crincoli, Andrea Ballini, Giorgia Apollonia Caloro, Lorenzo Lo Muzio

**Affiliations:** 1 Department of Clinical and Experimental Medicine, University of Foggia, Via Rovelli 50, 71122 Foggia, Italy; spirito.francesca97@gmail.com (F.S.);; 2 Department of Surgical Sciences, Dental School, University of Turin, 10127 Turin, Italy; mario.alovisi@unito.it; 3 Department of Biomedical, Surgical, and Dental Science, University of Milan, 20122 Milan, Italy; riccardo.aiuto@unimi.it; 4 Department of Dentistry, Universidad Europea de Valencia, Paseo de la Alameda 7, 46010 Valencia, Spain; daniele.garcovich@universidadeuropea.es; 5 Department of Basic Medical Sciences, Neurosciences and Sensory Organs, Division of Complex Operating Unit of Dentistry, “Aldo Moro” University of Bari, Piazza G. Cesare 11, 70124 Bari, Italy; vito.crincoli@uniba.it; 6 Department of Precision Medicine, University of Campania “Luigi Vanvitelli”, 80138 Naples, Italy; 7 Unità Operativa Nefrologia e Dialisi, Presidio Ospedaliero Scorrano, ASL (Azienda Sanitaria Locale) Lecce, Via Giuseppina Delli Ponti, 73020 Scorrano, Italy; giorgiacaloro1983@hotmail.it

**Keywords:** osteonecrosis, MRONJ, BRONJ, ONJ, antiresorptive drugs

## Abstract

In the treatment and prevention of osteoporosis and more generally of neoplastic and metabolic pathologies affecting bone tissues, antiresorption drugs such as bisphosphonates and monoclonal antibody are used. Bisphosphonates have been linked to cases of osteonecrosis of the jaws since 2003 by Marx, with more and more evidence over the next two decades; together with bisphosphonate drugs, cases relating to the use of monoclonal drugs have been subsequently added. Among the main independent risk factors, we have extraction procedures in oral surgery that can affect both the mandible and the maxilla and the anterior or posterior sectors. The incidence of MRONJ treated with oral bisphosphonates ranges from 0.5% to 3% according to studies; this incidence would appear to be higher in patients treated with antiresorptive agents with neoplastic diseases. Many pathologies including those in which antiresorptive drugs are used show differences in prevalence in relation to sex; similarly, there could be differences in the incidence of cases of osteonecrosis based on gender in patients undergoing dentoalveolar surgery. Therefore, the objective of this systematic review and trial sequential analysis was to identify and quantify whether there is a proportionally greater risk of MRONJ in male or female subjects and whether there is evidence of greater involvement of osteonecrosis at several extraction sites, differentiating them into mandibular or maxilla and in the anterior or posterior sector. The revision protocol followed the indications of the Cochrane Handbook, and were recorded in Prospero, while the drafting of the manuscript was based on PRISMA. The results of the systematic review, after the study identification and selection process, included a total of 24 studies. The results of the meta-analysis reports: odds ratio (random effects model): 1.476 (0.684, 3.184) between male and female; odds ratio (random effects model): 1.390 (0.801, 2.412) between mandible and maxillary, and an odds ratio value of 0.730 (0.250, 2.137) between the anterior and posterior extraction sites. In conclusion, we can see that there was a trend in the onset of MRONJ as a complication of dentoalveolar surgical procedures, which proportionally mostly involved the male sex and the posterior mandibular sectors, however, this trend must be further confirmed by additional studies.

## 1. Introduction

The bone tissue constantly undergoes a remodeling characterized by resorption and formation, in which a key role is played by two cell types such as osteoclasts and osteoblasts; these two moments are generally in balance in the adult, but with the advance in age, the number and activity of osteoblasts decrease while the action of osteoclasts increases.

Differentiation such as the maturation and activation of osteoclasts is programmed and influenced by osteoblasts through the expression of OPG (osteoprotegerin) and RANKL (receptor activator of nuclear factor kappa-B ligand) [1].

RANKL is expressed on the cytoplasmic surface by binding to its receptor in osteoclastic precursors; it stimulates hematopoietic cells, differentiating them into osteoclasts [2]. On the other hand, OPG is excreted by osteoblasts and leads to the inactivation of RANKL by preventing the differentiation and activation of osteoclasts [3].

This reabsorption mechanism, determined by an upregulation of RANKL, is widely demonstrated in menopausal female subjects suffering from osteoporosis [4]. Furthermore, bone loss in menopausal women is accelerated by the production of pro-inflammatory cytokines TNF-α, IL-6, and IL-1 [5]. The inflammation act plays a clear and evident role in bone resorption as occurs in periodontitis [6,7], peri-implantitis, and in healing tissue following tooth extractions.

In the treatment and prevention of osteoporosis, and more generally in neoplastic and metabolic pathologies involving bone tissues, antiresorptive drugs are used such as bisphosphonates (zoledronate, zoledronate, pamidronate, risedronate, ibandronate) [8] and the human monoclonal antibody: denosumab [9]. In addition, other antineoplastic monoclonal antibodies such as bevacizumab, sunitinib, and temsirolimus have also been associated with the development of osteonecrosis of the jaw (ONJ) [9].

The use of bisphosphonates in oncology is widely consolidated, and in some classes of carcinomas such as non TNBC or non HER2+ breast cancer [10], treatment with this class of drugs leads to a modification of the tumor microenvironment as well as a significant reduction in the mortality and recurrence rates [11].

Bisphophonates have been linked to cases of osteonecrosis of the jaw since 2003 by Marx with more and more evidence in the following two decades [12]; together with bisphosphonate drugs, cases were subsequently added to cases related to the use of monoclonal drugs, thus moving from a definition of BRONJ (bisphosphonate-related osteonecrosis of the jaw) [13] to that of MRONJ (medication-related osteonecrosis of the jaw) [14] and ARONJ (antiresorptive agent-related osteonecrosis of the jaw) [15].

Among the main independent risk factors, we have the extraction procedures in oral surgery [16]; in fact, extractions of the dental elements should be avoided if possible in patients with high dose therapies of antiresorptive agents [17].

The extractions of the dental elements are among the main surgical dental procedures and can affect both the mandible and the maxilla and the anterior sectors (incisors and canines) or the posterior sectors (molars and premolars) [18]. The different extraction sites have anatomical and tissue characteristics with different qualities and densities of the bone structures, which diversify the different extraction techniques [19,20].

The incidence of MRONJ treated with oral bisphosphonates ranges from 0.5% [21] to 3% [22] according to studies: the incidence would seem higher in patients treated with antiresorptive agents with neoplastic pathologies [23] while the incidence of MRONJ is higher in patients treated with antiresorptive agents for malignancy due to the fact that these agents are generally given IV in higher doses and with higher frequency [24].

Many pathologies including those in which antiresorptive drugs are used present differences in prevalence in relation to sex; similarly, there could be differences in the incidence of cases of osteonecrosis according to sex in patients undergoing dentoalveolar surgery [25].

The characteristics of the bone can vary in relation to the different anatomical positions. The bone remodeling processes following extractions undergo significant differences between the posterior and anterior regions, with greater evidence in the posterior sectors where the post-extraction atrophy of the mandible begins and progresses faster [26]. Moreover, at the level of the posterior maxilla, the remodeling and trabecular organization is more random, therefore, post-extraction bone resorption (which is inhibited by bisphosphonate drugs) could occur in a non-equal way in the maxillary bones, consequently, the characteristics of the bone in formation are influenced by the location in which they form [27].

Other evidence on a different bone characteristic comes from studies conducted by Mish [28], where in the posterior and maxillary sectors, there was the presence of bone with a thin porous layer and with fine trabecular bone (D3–D4), while there was denser bone in the anterior and posterior mandibles (D1–D2) [28].

These differences in bone histo-morphological composition are the reflection of a different bone remodeling process that occurs in these areas [29], differences that could be the cause of an altered incidence in the localization of MRONJ cases.

The knowledge of these differences by the oral surgeon who performs the tooth extractions can be clinically relevant in the choice of surgical technique. In fact, in these extraction sites, the execution of extraction techniques that are more respectful of the crestal bone as well as the use of suitable techniques in the preparation of a flap and sutures in the most difficult cases appear to be fundamental in the prevention of ONJ in patients taking drugs related to osteonecrosis.

Previous systematic reviews of the literature did not focus on the localization of MRONJ in relation to the extraction sites and gender. In fact, among the most recent, Schwech in 2020 [30] highlighted the incidence of cases of osteonecrosis in cancer patients; Aboalela et al. analyzed the aspects of the drug holiday in tooth extraction in patients treated with antiresorptive drugs in 2022 [31], and Cabras et al. in 2021 analyzed the possible efficacy of antibiotic therapy in the prevention of MRONJ during dentoalveolar surgery [32,33].

Therefore, the objective of this systematic review was to identify and quantify whether there is a proportionally greater risk of ONJ in male or female subjects, and whether there is evidence of a greater involvement of osteonecrosis in the different extraction sites, separating them into mandibular or maxillary and in the anterior or posterior sector.

## 2. Materials and Methods

### 2.1. Protocol and Registration

The systematic review was written following the PRISMA (preferred reporting items for systematic reviews and meta-analyses) guidelines [34]. All of the research, selection, and data extraction procedures followed the indications of the Cochrane Handbook, and the revision protocol was submitted and registered on the PROSPERO Platform with a registration number of CRD42023400788.

### 2.2. Eligibility Criteria

All prospective and retrospective studies and RCTs reporting data on the number of BRONJ, MRONJ, or ARONJ in patients who underwent dentoalveolar surgery and who made use of bisphosphonates and more generally of antiresorptive agents were considered potentially eligible.

In particular, studies were selected that reported the data on the osteonecrosis that occurred in the maxillary and mandibular surgical sites and in the anterior and posterior sectors, and further attention was paid to the differences in prevalence between the female and male sexes.

The PICO question formulated was therefore the following: whether there are differences in proportion in the onset of osteonecrosis in patients receiving the antiresorptive agent: in the surgical sites (between the mandibular and maxillary sectors, between the anterior and posterior sectors); between the female and male; (P)articipants (patients taking antiresorptive agents and undergoing dentoalveolar surgery); (I)ntervention (presence of osteonecrosis of the jaws in the different extraction sites), (C)ontrol (patients without osteonecrosis); and (O)utcome (odds ratio between the frequency of cases of osteonecrosis in the different surgical sites and between the sexes).

The inclusion criteria were as follows: studies reporting the data on the osteonecrosis experienced in patients using antiresorptive agents undergoing dentoalveolar surgery and reporting the location of the osteonecrosis (mandibular, maxillary, or anterior or posterior) or reporting the number of osteonecrosis between the two sexes.

The exclusion criteria were as follows: studies that did not report data on osteonecrosis cases or that only reported osteonecrosis cases, as a study population, studies published in a language other than English, and those at high risk of bias.

### 2.3. Sources of Information, Research and Selection

Studies were identified through literature searches of electronic databases by two authors (M.D. and A.B.). Publication language restrictions were enforced and non-English language articles were excluded. The literature search was conducted on the PubMed, Scopus, and Cochrane library databases. The last literature search was conducted on 16 February 2023. In addition, a gray literature search was also conducted by consulting Google Scholar, Science Direct, and Open Gray and the bibliographic sources of previous systematic reviews on the topic were also investigated.

We used the following terms to search the databases: BRONJ, MRONJ, Osteonecrosis, ARONJ, Bisphosphonates.

The following search terms were used on PubMed:

Search: BRONJ OR MRONJ OR Osteonecrosis jaw OR ARONJ OR Bisphosphonates osteonecrosis Sort by: Most Recent

“BRONJ” [All Fields] OR “MRONJ” [All Fields] OR ((“osteonecrosis” [MeSH Terms] OR “osteonecrosis” [All Fields] OR “osteonecroses” [All Fields]) AND (“jaw” [MeSH Terms] OR “jaw” [All Fields])) OR “ARONJ” [All Fields] OR ((“bisphosphonated” [All Fields] OR “bisphosphonic” [All Fields] OR “diphosphonates” [MeSH Terms] OR “diphosphonates” [All Fields] OR “bisphosphonate” [All Fields] OR “bisphosphonates” [All Fields]) AND (“osteonecrosis” [MeSH Terms] OR “osteonecrosis” [All Fields] OR “osteonecroses” [All Fields])).

Translations:

Osteonecrosis: [MeSH Terms] OR “osteonecrosis” [All Fields] OR “osteonecroses” [All Fields];

Jaw: “jaw” [MeSH Terms] OR “jaw” [All Fields];

Bisphosphonates: “bisphosphonated” [All Fields] OR “bisphosphonic” [All Fields] OR “diphosphonates” [MeSH Terms] OR “diphosphonates” [All Fields] OR “bisphosphonate” [All Fields] OR “bisphosphonates” [All Fields];

On the Scopus platform, instead, the following search terms and criteria were used:

TITLE-ABS-KEY (bronj OR mronj OR osteonecrosis AND jaw OR aronj OR bisphosphonates AND osteonecrosis).

Duplicates were removed using EndNote and manually. The identified articles were independently evaluated and reviewed by two reviewers (M.D. and A.B.), the evaluation of potentially eligible articles was carried out considering the title and abstract, while the full text was evaluated for inclusion in the systematic review. In addition, the *k* agreement between the two reviewers was assessed and a third reviewer resolved any disagreements.

### 2.4. Data Collection Process and Data Characteristics

The type of data and information to be extracted were previously determined by the two authors responsible for screening the articles and were independently transcribed into tables to be subsequently compared to minimize and reduce the risk of bias.

The data that were extracted from the articles concerned the first author, the year of publication, type of study, the country that conducted the study, the number of patients, the average age, the gender, the primary pathology for which antiresorptive agent was administered, the type of antiresorptive agent taken, the route of administration, the average duration of administration of the drug, the number of extraction sites or extracted teeth, the location of the surgical sites, the number of osteonecrosis and their location, and the distribution of cases between the sexes.

### 2.5. Risk of Bias within Individual Studies, Summary Measures, Summary of Results, Risk of Bias across Studies, Publication Bias and Additional Measures

The ROBINS-I tool was used to measure the risk of bias, and it was evaluated by the two authors (A.B. and M.D.) appointed to select the studies. The studies with a high risk of bias were excluded from the systematic review and meta-analysis.

The results were extracted and reported in tables while the aggregated data were represented in figures such as the forest plot with the respective numerical values of odds ratio (OdRa) and heterogeneity indices such as the Higgins index (*I*^2^).

The risk of bias between studies was assessed visually (funnel plot) by analyzing of the overlaps of the confidence intervals (C.I.), through the index of inconsistency *I*^2^ (a value of *I^2^* greater than 30% was considered medium and a random analysis was applied-effects in specific cases), and through a funnel chart. If the meta-analysis presented high indices of heterogeneity, a sensitivity analysis was performed excluding only the studies that presented a low overlap of the C.I. or that emerged graphically from the funnel plot.

For the meta-analysis, and in particular for the calculation of the pooled odds radio, the Reviewer Manager 5.4 software (Cochrane Collaboration, Copenhagen, Denmark), and Open Meta-Analyst version 10 were used. The GRADE pro-Guideline Development Tool online software (GRADE pro-GDT, Evidence Prime) and TSA (trial sequential analyses) using a Java-based software, the TSA software (Copenhagen Trial Unit, Center for Clinical Intervention Research, Copenhagen, Denmark) were also performed.

## 3. Results

### 3.1. Selection of Studies

The research question that guided the selection of the studies was as follows: whether there are proportional differences in the onset of osteonecrosis in patients treated with antiresorptive and anti-neoplastic agents at the surgical sites (between the mandibular and maxillary sectors, and/or between the anterior and posterior) and between the females and males.

The research phase was performed by consulting and extracting the bibliographic references on two databases, SCOPUS (7234 records) and PubMed (5036 records), and on a Cochrane Central Trials register (348 trials), providing a number of 12,618 records. The references were uploaded to EndNote X8 and the duplicates removed using software while the duplicates not identified by the software were identified manually and removed, obtaining a number of records equal to 7408.

**Figure 1:**
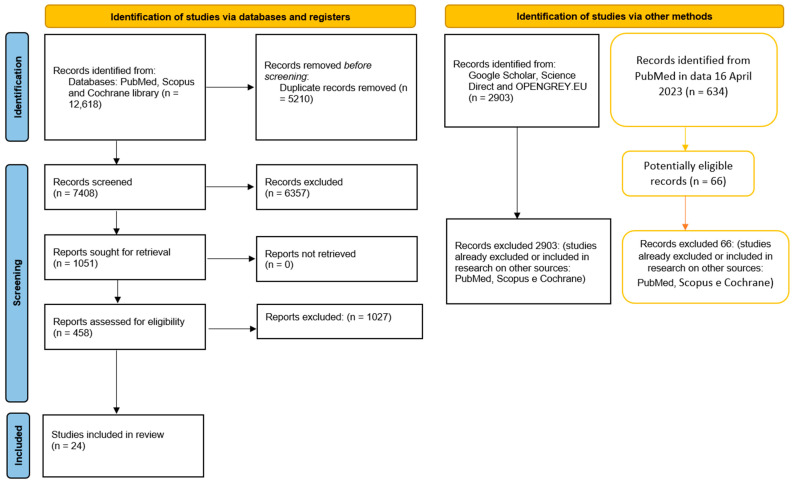
The entire selection and screening procedures are described in the PRISMA flowchart; tables with the orange lines are the searches performed subsequently (on 16 April 2022), with the addition of new keywords on PubMed.

After reading the record’s title and abstract, there reached an equal number of 458 items potentially eligible, and at the end of the selection, the articles included for the qualitative evaluation totaled 24. A further search of the gray literature (Google Scholar, Open Gray, and Science Direct) and previous systematic reviews was conducted that did not allow us to identify further studies to be included in the revision (Figure 1). The records were screened by two authors (M.D. and A.B.), independently, doubtful situations were addressed at the end of the selection involving a third author (FS) to resolve potential conflicts.

An update of the PubMed keywords was performed on 16 April 2023 with the addition of the following key words:

Search: denosumab AND osteonecrosis: (“denosumab” [MeSH Terms] OR “denosumab” [All Fields] OR “denosumab s” [All Fields]) AND (“osteonecrosis” [MeSH Terms] OR “osteonecrosis” [All Fields] OR “osteonecroses” [All Fields]); Translations: denosumab: “denosumab” [MeSH Terms] OR “denosumab” [All Fields] OR “denosumab’s” [All Fields];

Osteonecrosis: “osteonecrosis” [MeSH Terms] OR “osteonecrosis” [All Fields] OR “osteonecroses” [All Fields].

We obtained a number of records equal to 634, which were screened by the two authors in search of any clinical studies to be included; the results of this selection are highlighted in Figure 1.

### 3.2. Data Characteristics

The articles included in the review are as follows: Shudo et al. 2018 [35], Jeong et al., 2017 [36], Lain and Ajwani 2016 [37], Hasegawa et al., 2017 [22], Ferlito et al., 2011 [38], Hutcheson et al., 2014 [39], Lazarovici et al., 2010 [40], Lodi et al., 2010 [41], Migliorati et al., 2013 [42], Mozzati et al., 2013 [43], Mozzati et al., 2012 [44], Mozzati et al., 2011 [45], O’Connell et al., 2012 [46], Saia et al., 2010 [47], Scoletta et al., 2013 [48], Scoletta et al., 2011 [49], Vescovi et al., 2013 [50], Ottesen et al., 2021 [51], Kang et al., 2020 [52], Kawakita et al., 2017 [53], Fujieda et al., 2020 [54], Bodem et al., 2015 [55], O’Ryan and Lo 2012 [56], and Kunchur et al., 2009 [21].

The data extracted are reported in three tables. Table 1 represents the data concerning the first author, the country of the study, the type of study, the total number of patients, the average age or the range, the primary disease for which the drug is being administered, the type of drug administered, the route and duration of administration, and the number of extraction sites or extracted teeth.

The type of studies were heterogeneous: there were six retrospective studies and 13 prospective, to which must be added two case controls, one case series, one randomized study, and one non-randomized observational study. The total number of patients included taking antiresorptive drugs was 5817, of which 1106 were male (excluding studies of Ferlito et al. (2011) [38] and Lodi et al. (2010) [41], which did not provide indications on gender).

Table 2 shows the data relating to the number of extraction sites for the dentoalveolar surgery procedures and the number of patients as well as the number of MRONJs for the different extraction sites.

The total number of MRONJ in the included studies involving the male gender was 20 cases, while for the female gender, it was a total of 88 (Table 3).

### 3.3. Risk of Bias

The risk of bias was rated as acceptable for all studies included in the systematic review, but a more rigorous approach was taken in choosing to include the studies in the meta-analysis as well excluding some studies that reported non-homogeneous data or that, for example, concerned a panel of patients whose population of origin was not clearly defined and stratified, or not comparable with others (O’Ryan and Lo, 2012 [56]).

The studies were evaluated using the following parameters (ROBINS-I): due to confounding factors, bias in selection of participants into the study, bias in the classification of interventions, bias due to deviations from the intended interventions, bias due to missing data, bias in the measurement of outcomes, and bias in the selection of reported outcome; for each parameter, the assessment could be critical risk, severe risk, moderate risk, low risk or unmeasured risk (Table 4).

### 3.4. Meta-Analysis

The meta-analysis of the data was conducted using the Open Meta-Analyst version 10 (forest plot), and the Reviewer Manager 5.4 software (Cochrane Collaboration, Copenhagen, Denmark) for the construction of the funnel plot.

**Figure 2:**
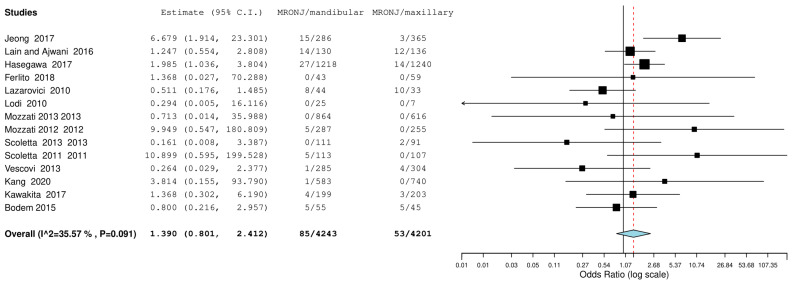
Binary random effects model metric; odds ratio: 1.390; C.I. (Confidence Interval): (lower bound) 0.801 (upper bound) 2.412; *p*-value 0.241; Q = Q statistic (measure of weighted squared deviations); df = degrees of freedom; I2 (I^2) = Higgins heterogeneity index, I2 < 50%, heterogeneity low; P = *p* value; heterogeneity (Het.): tau^2: 0.315; Q (df = 13) 20.178, Het. *p*-value: 0.091, I^2: 35.574; Results (log scale): 0.329 (−0.221, 0.880), Standard error (SE): 0.281; Weights: Jeong: 10.945%, Lain and Ajwani: 16.231%, Hasegawa: 18.572%, Ferlito: 1.814%, Lazarovici: 12.918%, Lodi: 1.760%, Mozzati 2013: 1.829%, Mozzati 2012: 3.154%, Scoletta 2013: 2.887%, Scoletta 2011: 3.140%, Vescovi: 5.024%, Kang: 2.646%, Kawakita: 8.692%, Bodem: 10.390%. Correction factor = 0.5 (applied only to values of 0). The graph of each study shows the first author and the date of publication as well as the measurement of the number of MRONJs on the total and the relative OdRa with the confidence intervals reported. The final value with the relative confidence intervals is expressed in bold. Jeong et al., 2017 [36], Lain and Ajwani, 2016 [37], Hasegawa et al., 2017 [22], Ferlito et al., 2011 [38], Lazarovici et al., 2010 [40], Lodi et al., 2010 [41], Mozzati et al., 2013 [43], Mozzati et al., 2012 [44], Scoletta et al., 2013 [48], Scoletta et al., 2011 [49], Vescovi et al., 2013 [50], Kang et al., 2020 [52], Kawakita et al., 2017 [53], Bodem et al., 2015 [55].

The first meta-analysis conducted concerned the mandibular or maxillary localization of the MRONJ, and random effects were applied according to DerSimonian and Laird by calculating the OdRa (the probability that the MRONJ occurs in the mandibular site compared to the probability that the MRONJ occurs in the maxillary site). The value of the odds ratio turned out to be slightly in favor of a smaller number of probabilistic events in the maxillary area—OdRa: 1.390 C.I. (0.801, 2.412), *p* value 0.241, however, the central rhombus that gives the size of the effect intercepted the central line of no effect (Figure 2).

**Figure 3:**
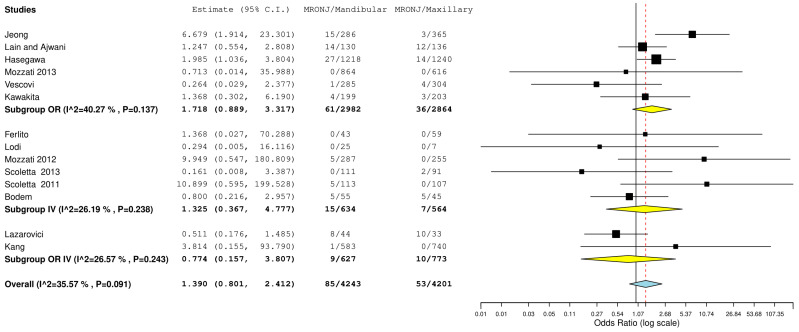
Forest plot analysis subgroup; subgroup OR: 6 studies, OdRa: 1.718 (0.889, 3.317), SE: 0.336, *p*-Val: 0.107, z-Val: 1.611, Q (df): 8.371 (5), Het. *p*-Val: 0.137, I^2: 40.27%; Subgroup IV: 6 studies, OdRa: 1.325 (0.367, 4.77), SE: 0.654, *p*-Val: 0.667, z-Val: 0.430, Q (df): 6.774 (5), Het. *p*-Val: 0.238, I^2: 26.19%; Subgroup OR IV: 2 studies, OdRa: 0.774 (0.157, 3.807), SE: 0.813, *p*-Val: 0.752, z-Val: −0.316, Q (df): 1.362 (1), Het. *p*-Val: 0.243, I^2: 26.57%. Jeong et al., 2017 [36], Lain and Ajwani, 2016 [37], Hasegawa et al., 2017 [22], Ferlito et al., 2011 [38], Lazarovici et al., 2010 [40], Lodi et al., 2010 [41], Mozzati et al., 2013 [43], Mozzati et al., 2012 [44], Scoletta et al., 2013 [48], Scoletta et al., 2011 [49], Vescovi et al., 2013 [50], Kang et al., 2020 [52], Kawakita et al., 2017 [53], Bodem et al., 2015 [55].

An analysis of subgroups was also conducted according to the route of administration of the antiresorptive drug adopted in the studies, whether intravenous (IV) or oral (OR), or whether they included patients whose therapy could be either IV or OR. The value of the odds ratio aggregated for the subgroups depicted in Figure 3 did not deviate in individual values from the overall odds ratio given by the inclusion of patients from all studies.

**Figure 4:**
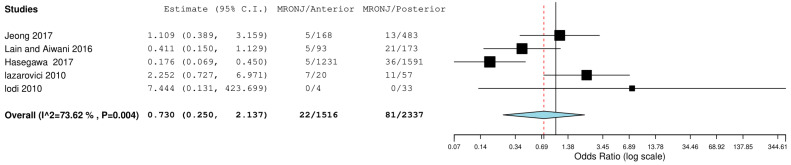
Binary random effects model: OdRa: 0.730 (0.250, 2.137), *p*-value: 0.566, tau^2: 0.996, Q (df = 4): 15.165, Het. *p*-value: 0.004, I^2: 73.624. Results (log scale) −0.314 (−1.388, 0.760) SE: 0.548. weights: Jeong: 23.420%, Lain and Aiwani: 23.784%, Hasegawa: 24.489%, Lazarovici: 22.588%, Lodi: 5.718%. Jeong et al., 2017 [36], Lain and Ajwani, 2016 [37], Hasegawa et al., 2017 [22], Lazarovici et al., 2010 [40], Lodi et al., 2010 [41].

The second meta-analysis was performed including data from studies that reported information on the location of the MRONJ (anterior or posterior); the data in the meta-analysis reported an OdRa value of 0.730 (0.250 2.137), slightly in favor of a minor involvement of the anterior sectors by osteonecrosis. Additionally in this case, as graphically deduced from the forest plot (Figure 4), the central rhombus intercepted the central line of the non-effect.

**Figure 5:**
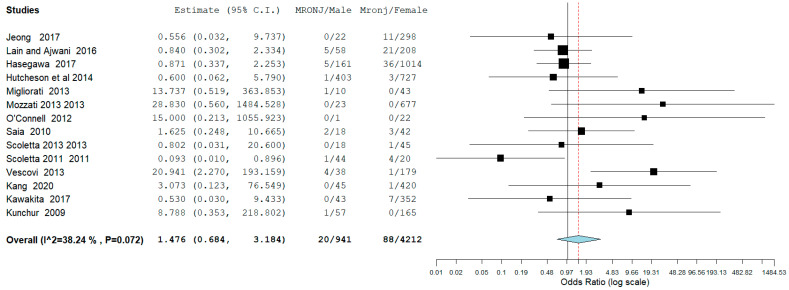
Binary random effects model: OdRa: 1.476 (0.684, 3.184), *p*-value: 0.321, tau^2: 0.692, Q (df = 13): 21.049, Het. *p*-value: 0.072, I^2: 38.239. Results (log scale) 0.389 (−0.380, 1.158) SE: 0.392; Weights: Jeong: 5.444%, Lain and Ajwani: 15.968%, Hasegawa: 16.599%, Hutcheson et al.: 7.585%, Migliorati: 4.414%, Mozzati 2013: 3.250%, O’Connell: 2.849%, Saia: 9.539%, Scoletta 2013: 4.480%, Scoletta 2011: 7.590%, Vescovi: 7.785%, Kang: 4.549%, Kawakita: 5.398%, Kunchur: 4.550%. Jeong et al., 2017 [36], Lain and Ajwani, 2016 [37], Hasegawa et al., 2017 [22], Hutcheson et al., 2014 [39], Migliorati et al., 2013 [42], Mozzati et al., 2013 [43], O’Connell et al., 2012 [46], Saia et al., 2010 [47], Scoletta et al., 2013 [48], Scoletta et al., 2011 [49], Vescovi et al., 2013 [50] Kang et al., 2020 [52], Kawakita et al., 2017 [53], Kunchur et al., 2009 [21].

The result of the third meta-analysis concerned the probability (odds ratio) that MRONJ occurs in the male gender compared to the probability that MRONJ occurs in the female gender. The meta-analysis was slightly in favor for females—OdRa: 1.476 (0.684, 3.184), with fewer events in proportion to the male gender (Figure 5).

### 3.5. Risk of Bias across Studies: Publication Bias

**Figure 6:**
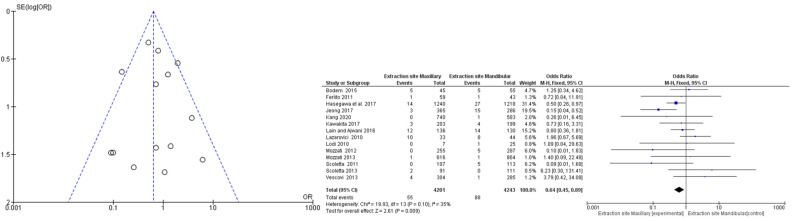
Funnel plot and forest plot (RevManger 5.4): OR, odds ratio; SE, standard error. Graphically, there are no sources of heterogeneity. The odds ratio value mirrors Figure 2, a correction factor d of 1 was applied to studies with mandibular and maxillary MRONJ events equal to 0. Jeong et al., 2017 [36], Lain and Ajwani, 2016 [37], Hasegawa et al., 2017 [22], Ferlito et al., 2011 [38], Lazarovici et al., 2010 [40], Lodi et al., 2010 [41], Mozzati et al., 2013 [43], Mozzati et al., 2012 [44], Scoletta et al., 2013 [48], Scoletta et al., 2011 [49], Vescovi et al., 2013 [50], Kang et al., 2020 [52], Kawakita et al., 2017 [53], Bodem et al., 2015 [55].

For the first meta-analysis, the publication bias was evaluated through the graphical construction of a funnel plot using the Reviewer Manager 5.4 software program (Cochrane Collaboration, Copenhagen, Denmark), resulting in the distribution of the 14 studies seeming to be homogeneous (Figure 6). Furthermore, the absence of sources of heterogeneity was highlighted, which confirmed a low heterogeneity of the studies as graphically evidenced by the overlapping of the confidence intervals, the heterogeneity values, and by the value of the Higgins index I2: 35 (Figure 6).

### 3.6. Trial Sequential Analysis: Grade

**Figure 7:**
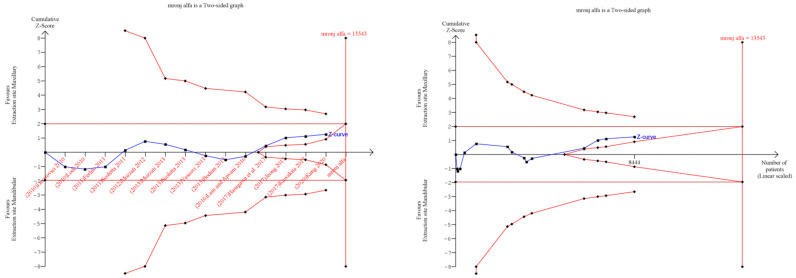
TSA: Red lines represent the sequential trial monitoring limits and futility limits. The solid blue line is the cumulative Z-curve that requires the information dimension to demonstrate or reject a 20% relative increase in benefit at the maxillary versus mandibular extraction site (5% alpha and 80% beta), whose results included 13,543 patients (vertical red line). The cumulative Z-curve not crossing the Z line (horizontal red line), Z = 1.96, indicates an absence of evidence because the meta-analysis included fewer patients than the required information size, which is a false negative result. Jeong et al., 2017 [36], Lain and Ajwani, 2016 [37], Hasegawa et al., 2017 [22], Ferlito et al., 2011 [38], Lazarovici et al., 2010 [40], Lodi et al., 2010 [41], Mozzati et al., 2013 [43], Mozzati et al., 2012 [44], Scoletta et al., 2013 [48], Scoletta et al., 2011 [49], Vescovi et al., 2013 [50], Kang et al., 2020 [52], Kawakita et al., 2017 [53], Bodem et al., 2015 [55].

Trial sequential analysis (TSA) was executedto estimate the potency of the result of the first outcome, and by adjusting the results to avoid type II and I errors. The program used was TSA free software. The O’Brien–Fleming spending function was utilized by applying random effects; for the purpose of determining the optimal sample size and for the power of the results, a RRR (relative risk reduction) of 20%, an alpha value of 5% (type 1 error), and a beta value of 80% (type 2 error) were used (Figure 7).

The authors also used GRADE pro-GDT to assess the quality of the evidence on the outcome. The results suggest that the quality of evidence is low (Table 5).

## 4. Discussion

The authors performed a systematic review of the literature, in order to ascertain whether there were differences in the gender and localization of osteonecrosis of the jaws from bone antiresorptive drugs following dentoalveolar surgical procedures. The present systematic review is currently the first review with meta-analysis and trial sequential analyses to assess the power of the findings of the meta-analysis conducted on these specific outcomes. The review work involved 14 articles and the total number of patients included in this study was 5817, of which 1106 were male.

The first report concerning osteonecrosis of the jaw as a complication of tooth extractions in patients taking antiresorptive drugs came from Marx et al. and it was clear that tooth extraction became the main trigger for osteonecrosis [12]. From subsequent studies, it became evident that osteoporotic patients, who were mainly female, were largely involved by MRONJ. Besides being involved with osteonecrosis of the jaw, there are also cancer patients (prostatic carcinoma, breast carcinoma, multiple myeloma, lung carcinoma, ovarian carcinoma, oral cancer) including those of the male sex, and it was strongly advised that the prevention of osteonecrosis should be performed by extracting compromised and no longer recoverable teeth before starting drug therapies [57].

With the emergence of new antiresorptive drugs, it is important to give clear answers on which kinds of patients are at risk of osteonecrosis complications as well as the related surgical extraction sites together with the procedures that may be mainly involved in this complication [58].

The aspects related to the age of MRONJ onset were extensively investigated by a very recent systematic literature review conducted by Rosales et al. (2023) [59] where it was concluded that there was a low presence of MRONJ in the infantile and juvenile population treated with antiresorptive drugs [59,60], while the incidence in patients with cancer receiving high doses of antiresorptive drugs was investigated by Schwech et al. [30].

The aspect of a drug holiday in the onset of osteonecrosis following dentoalveolar surgery was not covered in this meta-analysis as it was performed and extensively discussed by Aboalela et al. (2022) [31] and Ottesen et al. (2020) [61], who stated the absence of high-level evidence for the use of the drug holiday, mainly due to the high heterogeneity of the patients and therapies in the included studies.

From a previous systematic review in 2015 with the meta-analysis of data (comparison between cancer patients and osteoporotic patients) conducted by Gaudin [62], data on the differences between the two sexes and osteonecrosis localization was reported, with eight cases of MRONJ in male patients, and 13 for the females, presenting a number of patients included equal to 2566 (females 2098, males 468), with 13 presenting mandibular, and 23 maxillary localizations. No data were reported on the localization of osteonecrosis at the anterior or posterior extraction sites [62].

The data emerging from this meta-analysis reported a number of MRONJ in patients undergoing dentoalveolar surgery equal to 20 out of 941 for males and 88 out of 4212 for females with an aggregate OdRa (random effects model): 1476 (0.684, 3.184) (Figure 5), slightly in favor for the female sex (smaller number of MRONJ in proportion). For the localization of osteonecrosis, the reported data were 85 MRONJ following 4243 mandibular extraction sites, and 53 MRONJ following of 4202 maxillary extraction sites with the aggregate OdRa (random effects model) of 1.390 (0.801, 2.412) (Figure 2), reporting 22 MRONJ after 1516 anterior sector extractions, and 81 MRONJ after 2337 posterior sector extractions, the aggregate OdRa (random effects model) was 0.730 (0.250, 2.137) (Figure 4), with values slightly in favor for a location in the maxillary and anterior sites (favor: with proportionally fewer cases of MRONJ).

The subgroup analysis differentiating between the routes of administration did not reveal substantial differences between the locations of the MRONJ (Figure 3). Furthermore, the TSA (Figure 7) indicated that assuming an RRR equal to 20% between the maxillary and mandibular sites, the optimal number of patients was not reached.

The biological rationale for which the BRONJ, and more generally the MRONJ, is more probable (in proportion) in male subjects who undergo tooth extractions must be sought in the nature of the primary pathologies [63].

In the males, patients taking drugs related to osteonecrosis, bisphosphonates, denosumab, and antineoplastic drugs (bevacizumab, sunitinib, temsirolimus [64]) were generally subjects with malignancies who took intravenous drugs at high doses, while for the female sex, in the majority of cases, oral antiresorptive drugs were administered for the treatment and prevention of osteoporosis [65].

These two conditions, the tumor and osteoporotic scenarios, are likely to determine the differences in the proportion of the tendency in the onset of osteonecrosis in the two genders in patients undergoing dentoalveolar procedures [66].

The results of our meta-analysis partially agreed with the data of Suryani et al. [66] in cases of osteonecrosis of the jaw in patients taking non-resorptive drugs.

These authors identified a total of 867 patients with MRONJ (33% female, 55% male, 12% unspecified) in the literature, in which the mandibular region developed the greatest number of osteonecrosis (35%), followed by the maxilla (14%) [66].

The more compact nature of the bone (D1, D2), could lead to greater cases of osteonecrosis in the posterior mandibular sectors, which could lead to greater bone trauma during tooth extraction, if techniques that protect the alveolar bone are not adopted. Furthermore, bone remodeling (inhibited by antiresorptive agents) as well as edentulous ridge atrophy occur more quickly in the posterior mandibular sector following extraction.

Among the limitations of the review is the heterogeneity of the included studies, which were non-randomized retrospective; additionally, we confirm that most of the studies presented a small population sample size. Finally, the GRADE evaluation reported a low result, therefore the results of the meta-analysis outlining a trend should be considered with the proper limitation.

## 5. Conclusions

In conclusion, we can evaluate that within the limits of the systematic review and meta-analysis, there is a trend in the onset of MRONJ as a complication of dentoalveolar surgical procedures, which proportionally mostly involves the male sex and the posterior mandibular sector. However, this trend must be further confirmed by further studies as emerges from the TSA.

## Figures and Tables

**Table 1:** Characteristics of the studies included in the systematic review; the main characteristics of the groups of patients included in the studies are also reported, F (female), M (male), DS (deviation standard), Y (years), m (month), OR (oral administration), IV (intravenous administration), BF (bisphosphonates), RTC (randomized controlled trial), retrospective study (RS), retrospective multicenter study (RMS), prospective study (PS), case-control study (CCS), case series (CS), observational longitudinal noncontrolled study (OLNS), \ data not present or not reportable, ^1^ A total of 184 teeth in 102 extraction sites, ^2^ The number of extraction sites is not specified, ^?^ data reported but not clearly specified in the study.

First Autor, Data	Country	Study Design	Population (F, M)	Mean Age (y), DS, Range Age (y)	Primary Disease	Type of Administration (OR, IV)	Duration of Administration, Mean DS (m), Range (m)	Extraction\Procedure Site
Shudo et al., 2018 [35]	Japan	PS	132 (112, 20)	71.9 ± 11.4, (40–94)	Primary osteoporosis, prevention osteoporosis	OR: Alendronate (59), Risedronate (37), Minodronate (31), Ibandronato (5).	40.4 ± 38.0, 1–162	274
Jeong et al., 2017 [36]	Korea	PS	320 (298, 22)	111 patients < 65 y	Osteoporosis	OR: Alendronate (161) Risedronate (73), Ibandronato (20).	140 patients < 3 y	651
Lain and Ajwani, 2016 [37]	Australia	RS	266 (OR) (208, 58)	73.3 ± 6.9	Osteoporosis and cancer	OR: 266 Alendronate (203), Risedronate (55), Etidronate (1) Conbination (3), Unknown (4) IV:9	\	266
Hasegawa et al., 2017 [22]	Japan	RMS	1175 (1014, 161)	70.7 ± 11.7 (23–102)	Osteoporosis and cancer	OR: Alendronate (695), Risedronate (304), Minodronate (106), Others (8), Alendronate/Risedronate (27), Alendronate/Minodronate (19), Alendronate/Others (1), Risedronate/Minodronate (3), Minodronate/Others (1), Unknown (11).	38.5 ± 37.7, 1–246	2458
Ferlito et al., 2011 [38]	Italy	OLNS	43	56.4 ± 5.8	Multiple myeloma, breast cancer, prostate cancer, lung cancer	IV: Zolendronato	16.2 ± 3.2	102
Hutcheson et al., 2014 [39]	Australia	PS	950 (727, 403)	71	Osteoporosis	OR: Alendronate (560) Risedronate (373), Other combinations (17).	199 patients >5 y	2461
Lazarovici et al., 2010 [40]	Israel	PS	78 (63, 15)	F 64.2, (20–89); M 62.63, (9–81).	Osteoporosis, breast carcinoma, multiple myeloma, prostate carcinoma, neurogenic carcinoma	OR: Alendronate (44), Risedronate (3), Zoledronic acid (10), Pamidronate (7). IV: Alendronate and Risendronate (4), Zoledronic acid and Clodronate (2), Pamidronate and Clodronate (1).	Or: 42–144, IV: 24–61	78
Lodi et al., 2010 [41]	Italy	PS	23	68.2, (44–83)	Multiple myeloma, bone metastasis of breast cancer or other solid tumors and severe osteoporosis	IV: Zoledronate (20), Pamidronate (2), Clodronate (1).	17.5, 3–36	38
Migliorati et al., 2013 [42]	Canada, Norway, USA	PS	53 (43, 10)	70, (40–92)	Osteoporosis metastatic bone cancer	IV 13\45, OR 32\45	60	53
Mozzati et al., 2013 [43]	Italy	PS	700 (677, 23)	(52–79)	Osteoporosis, rheumatoid arthritis, and Paget’s disease.	OR: Alendronate	\	\
Mozzati et al., 2012 [44]	Italy	CCS	176 (101, 75)	(44–83)	Prostatic carcinoma, breast carcinoma, multiple myeloma, lung carcinoma, ovarian carcinoma	IV: Zoledronic acid	\	\
Mozzati et al., 2011 [45]	Italy	CCS	100 (75, 25)	(44–83)	Prostatic carcinoma, breast carcinoma, multiple myeloma, lung carcinoma, ovarian carcinoma	IV: Zoledronic acid (53), Pamidronate (47)	\	\
O’Connell et al., 2012 [46]	Ireland	PS	23 (22, 1)	59, (44–78)	Osteoporosis	OR: Acid Alendronic (19), Risendronate (2); IV: Zoledronic Acid (2)	30 (8–72)	23 ^?^
Saia et al., 2010 [47]	Italy	PS	60 (42, 18)	65 ± 13, (17–84)	Cancer	IV: Zoledronate (38), Pamidronate (24), Neridronate (4), OR: Risedronate (2);	\	185
Scoletta et al., 2013 [48]	Italy	PS	63 (45, 18)	65.82 ± 8.82	Cancer and osteoporosis	IV: Zoledronic acid (54), Pamidronate (4), Ibandronate (5)	19.03 months	202
Scoletta et al., 2011 [49]	Italy	PS	64 (44, 20)	64.81 ± 10.98	Cancer and osteoporosis	IV: Zoledronic acid (57), Pamidronate (2), Zoledronic acid + Pamidronate (5)	16.20	220
Vescovi et al., 2013 [50]	Italy	CS	217 (179, 38)	68.72, (30–83)	Cancer and osteoporosis	Zoledronate (87), Zoledronate + Pamidronate (1), Alendronate 54, Risedronate 18, Alendronate + Zoledronate (3), Clodronate (24), in 30 cases different association of BPs.	17 (cancer) 53 (osteoporosis)	589
Ottesen et al., 2021 [51]	Denmark	RTC	23 (12, 11)	69 (59–77), 67 (56–78)	Cancer	IV: Denosumab (13), Bisphosphonate (10).	9 (range 2–30) 17.5 (range 4–96)	31
Kang et al., 2020 [52]	Korea	RS	465 (420, 45)	M 3.7 ± 10.5; F 69.3 ± 8.8	Osteoporosis and cancer	OR: 410 Alendronate IV and OR: 30 Ibandronate and Alendronate. IV: Ibandronate 26	OR: 39.0 ± 35.5; IV:(40.0 ± 35.6)	1323
Kawakita et al., 2017 [53]	Japan	RS	341 (352, 43)	72.4 ± 10.6, 74.1 ± 9.62	Osteoporosis and cancer	OR	43.4 ± 36.3; 31.3 ± 31.0	850
Fujieda et al., 2020 [54]	Japan	RS	232 (202, 30)	71 (24–94)	Autoimmune disease	Alendronate (111), Risedronate (80), Minodronate (23), Ibantronate (4), Denousumab (14)	37 (17–51)	\
Bodem et al., 2015 [55]	Germany	PS	61 (42, 19)	65.65 ± 12.69 (34–87)	Cancer	IV: Zoledronic acid (38), Ibandronate (17), and Pamidronate (6).	40.25 ± 32.91; (4–245)	102 (184) ^1^
O’Ryan and Lo, 2012 [56]	USA	RS	30 (26, 4)	(54–89)	Osteoporosis	\	\	\
Kunchur et al., 2009 [21]	Australia	PS	222 (165, 57)	OR 71 ± 11.6, IV 61 ± 11	Osteoporosis and cancer	OR: Alendronate (139) Risedronate (76); IV: Pamidronate (6), Zoledronic acid (1)	OR: 50.5 ± 32 (2–180) IV: 26.9 ± 25 (21–72)	194 procedure and 21 endodontic therapy ^2^

**Table 2:** Number of MRONJ events that occurred in the different extractive sites. \ Data not present or not reportable, F (female), M (male), ^1^ One patient underwent surgery in both jaws. ^2^ Two patients involved both jaws, ^3^ Ten sites in eight patients, ^?^ data reported but not clearly specified in the study.

First Autor, Data	Population (F, M)	Extraction Site Total	Extraction Site Maxillary\MRONJ Site	Extraction Site Mandibular\MRONJ	Extraction Site Anterior\MRONJ	Extraction Site Posterior\MRONJ	MRONJ Total
Shudo et al., 2018 [35]	132 (112, 20)	274	165\0	109\0	97	177	0
Jeong et al., 2017 [36]	320 (298, 22)	651	365\3	286\15	168\5	483\13	11 patients, 18 sites
Lain and Ajwani 2016 [37]	266 (208, 58)	266	136\12	130\14	93\5	173\21	26 sites
Hasegawa et al., 2017 [22]	1175 (1014, 161)	2458	1240\14	1218\27	1231\5	1591\36	41 sites
Ferlito et al., 2011 [38]	43	102	59\0	43\0	\	\	102 sites
Hutcheson et al., 2014 [39]	950 (727, 403)	2461	\	\	\	\	4
Lazarovici et al., 2010 [40]	78 (63, 15)	78	33 ^1^\10	44\8	20\7	57\11	18 patients
Lodi et al., 2010 [41]	23 (15, 8)	38	7 ^2^\0	25\0	4\0	33\0	0
Migliorati et al., 2013 [42]	53 (43, 10)	53	\	\	\	\	1
Mozzati et al., 2013 [43]	700 (677, 23)	1480	616	864	\	\	0
Mozzati et al., 2012 [44]	176 (101, 75)	542	255\0	287\5	\	\	5
Mozzati et al., 2011 [45]	100	222	108	114	\	\	2
O’Connell et al., 2012 [46]	23 (22, 1)	23 ^?^	\	\	\	\	0
Saia et al., 2010 [47]	60 (42,18)	185	82	103	\	\	5 patients
Scoletta et al., 2013 [48]	63 (45, 18)	202	91\2	111\0	\	\	2
Scoletta et al., 2011 [49]	64 (44, 20)	220	107\0	113\5	\	\	5
Vescovi et al., 2013 [50]	217 (179, 38)	589	304\4	285\1	\	\	5
Ottesen et al., 2021 [51]	23 (12, 11)	31	18	13	\	\	4
Kang et al., 2020 [52]	465 (420, 45)	1323	740\0	583\1	\	\	1
Kawakita et al., 2017 [53]	341 (352, 43)	850	203\3	199\4	\	\	7
Fujieda et al., 2020 [54]	232 (202, 30)	\	\	\	\	\	10
Bodem et al., 2015 [55]	61 (42, 19)	102	45\5	55\5	\	\	10 (8 ^3^)

**Table 3:** Number of MRONJ in the male and female sex; \ Data not present or not reportable.

First Autor, Data	Male (MRONJ)	Male Total	Female (MRONJ)	Female Total
Shudo et al., 2018 [35]	0	20	0	212
Jeong et al., 2017 [36]	0	22	11	298
Lain and Ajwani, 2016 [37]	5	58	21	208
Hasegawa et al., 2017 [22]	5	161	36	1014
Ferlito et al., 2011 [38]	\	\	\	\
Hutcheson et al., 2014 [39]	1	403	3	727
Lazarovici et al., 2010 [40]	\	15	\	63
Lodi et al., 2010 [41]	0	\	0	\
Migliorati et al., 2013 [42]	1	10	0	43
Mozzati et al., 2013 [43]	0	23	0	677
Mozzati et al., 2012 [44]	\	75	\	101
Mozzati et al., 2011 [45]	\	\	\	\
O’Connell et al., 2012 [46]	0	1	0	22
Saia et al., 2010 [47]	2	18	3	42
Scoletta et al., 2013 [48]	0	18	1	45
Scoletta et al., 2011 [49]	1	44	4	20
Vescovi et al., 2013 [50]	4	38	1	179
Ottesen et al., 2021 [51]	\	11	\	12
Kang et al., 2020 [52]	0	45	1	420
Kawakita et al., 2017 [53]	0	43	7	352
Fujieda et al., 2020 [54]	\	30	\	202
Bodem et al., 2015 [55]	\	19	\	42
Kunchur et al., 2009 [21]	1	57	0	165
O’Ryan and Lo, 2012 [56]	\	4	\	26

**Table 4:** Risk of bias: low risk +, moderate risk -, serious risk x, critical risk !, unmeasured risk ?.

First Autor, Data	Bias Due to Confounding	Bias in Selection of Participants into the Study	Bias in Classification of Interventions	Bias Due to Deviations from Intended Interventions	Bias Due to Missing Data	Bias in Measurement of Outcomes	Bias in Selection of the Reported Result	
Shudo et al., 2018 [35]	+	+	+	+	+	+	+	+
Jeong et al., 2017 [36]	-	+	+	+	+	+	+	+
Lain and Ajwani, 2016 [37]	+	+	+	+	+	+	+	+
Hasegawa et al., 2017 [22]	+	+	+	+	+	+	+	+
Ferlito et al., 2011 [38]	+	+	+	+	?	+	?	+
Hutcheson et al., 2014 [39]	+	+	+	+	?	+	+	+
Lazarovici et al., 2010 [40]	+	+	+	+	+	+	+	+
Lodi et al., 2010 [41]	+	+	+	+	+	+	+	+
Migliorati et al., 2013 [42]	+	+	?	+	?	+	?	+
Mozzati et al., 2013 [43]	+	+	?	+	?	+	?	+
Mozzati et al., 2012 [44]	+	+	+	+	?	+	+	+
Mozzati et al., 2011 [45]	+	+	+	+	?	+	?	+
O’Connell et al., 2012 [46]	+	+	+	+	?	+	?	+
Saia et al., 2010 [47]	+	+	+	+	?	+	?	+
Scoletta et al., 2013 [48]	+	+	+	+	?	+	?	+
Scoletta et al., 2011 [49]	+	+	+	+	?	+	?	+
Vescovi et al., 2013 [50]	+	+	+	+	?	+	?	+
Ottesen et al., 2021 [51]	+	+	+	+	+	+	+	+
Kang et al., 2020 [52]	+	+	+	+	+	+	+	+
Kawakita et al., 2017 [53]	+	+	+	+	?	+	?	+
Fujieda et al., 2020 [54]	+	+	+	+	?	+	?	+
Bodem et al., 2015 [55]	+	+	+	+	?	+	?	+
Kunchur et al., 2009 [21]	+	+	+	+	?	+	+	+
O’Ryan and Lo, 2012 [56]	+	-	+	+	?	+	+	+

**Table 5:** Evaluation of GRADE pro GDT: ⊕◯◯◯ Very low, ⊕⊕◯◯ Low, ⊕⊕⊕◯ Moderate, ⊕⊕⊕⊕ High.

Certainty Assessment							No. of Patients		Effect		Certainty
No. of studies	Study design	Risk of bias	Inconsistency	Indirectness	Imprecision	Other considerations	MRONJ Maxillary	MRONJ Mandibular	Relative (95% CI)	Absolute (95% CI)	
14	Observational studies	Not serious	not serious	not serious	not serious	none	55/4201 (1.4%)	88/4243 (2.1%)	OR 0.68 (0.48 to 0.95)	7 fewer per 1.000 (from 11 fewer to 1 fewer)	⨁⨁◯◯ Low

## Data Availability

Not applicable.
